# Esophagus perforation and myocardial penetration caused by swallowing of a foreign body leading to a misdiagnosis of acute coronary syndrome: a case report

**DOI:** 10.1186/s13256-015-0532-9

**Published:** 2015-03-12

**Authors:** Uysal Erdal, Dokur Mehmet, Kirdak Turkay, Ikidag A Mehmet, Nacak Ibrahim, Bakir Hasan

**Affiliations:** 1 Department of General Surgery and Transplantation Center, Sanko University Hospital, Gaziantep, Turkey; 2 Sanko University Hospital, Department of Emergency, Gaziantep, Turkey; 3 Uludag University School of Medicine, Department of General Surgery, Bursa, Turkey; 4 Department of Radiology, Sanko University Hospital Gaziantep, Gaziantep, Turkey; 5 Sanko University Hospital, Department of Thoracic Surgery, Gaziantep, Turkey; 6 Department of General Surgery, Sanko University Hospital, Gaziantep, Turkey

**Keywords:** Acute coronary syndrome, Esophagus perforation, Foreign body

## Abstract

**Introduction:**

Here we present our clinical experience in a case of esophagus perforation due to the swallowing of a bone piece causing acute angina pectoris and leading to misdiagnosis of acute coronary syndrome.

**Case presentation:**

A 73-year-old Caucasian man underwent urgent coronary angiography with possible diagnosis of acute coronary syndrome. His coronary arteries were found to be normal. A computed tomography examination revealed esophagus perforation by a foreign body (a piece of bone), and he underwent urgent left thoracotomy and the foreign body was removed.

**Conclusions:**

Sometimes, even a piece of bone within a meal can lead to esophagus perforation, and injure the pericardium and myocardium. The symptoms of esophagus perforation may be confused with acute coronary syndrome due to their similarities and lack of knowledge about the detailed clinical history as shown in our case. Thus, careful consideration of detailed clinical history as well as choosing an appropriate medical imaging modality, such as computed tomography, should always be kept in mind in order to promptly diagnose and start early treatment to reduce mortality.

## Introduction

Esophagus foreign bodies are mostly encountered in childhood. Although they are rarely seen in adults, the rate of mortality and morbidity is high in cases of perforation. Most esophagus perforations are iatrogenic [1]. Perforations due to foreign bodies are rare, and complications are mostly encountered with sharp-edged objects [2]. Foreign bodies in the esophagus should be removed immediately because of the risk of complications [3]. A few cases of cardiac tamponade due to esophagus perforation have been reported, but the symptoms were not confused with acute coronary syndrome [2,3]. Esophagus perforation should be kept in mind in patients referring with acute chest pain. Although clinical history and radiological evaluation are usually sufficient for the diagnosis, computed tomography (CT) is the most sensitive diagnostic modality. Patients may refer with shortness of breath and chest pain. Prompt diagnosis and early treatment are important in preventing high mortality and morbidity rate in cases of esophagus perforation [4]. Early surgery should be the first treatment choice. Here we present our clinical experience in a case of esophagus perforation due to swallowing of a bone piece causing acute angina pectoris and leading to misdiagnosis of acute coronary syndrome.

## Case presentation

**Figure 1:**
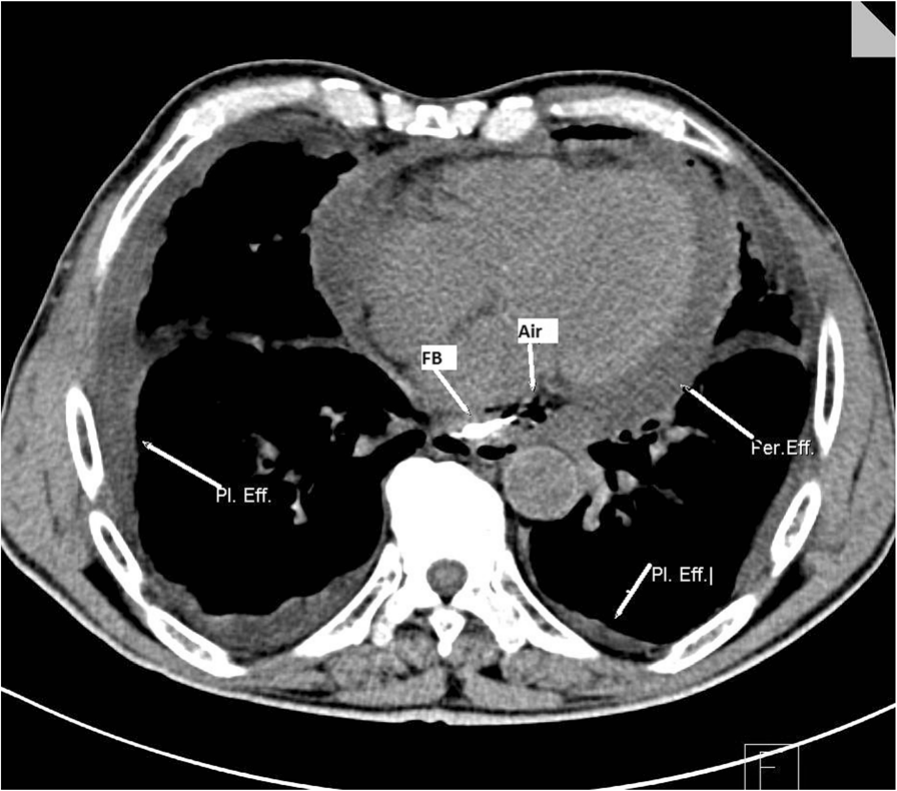
**Findings of esophageal perforation by foreign body on computed tomography.** Abbreviations: FB, Foreign body; Per. Eff., Pericardial effusion; Pl. Eff., Pleural effusion.

A 73-year-old Caucasian man was referred to another health care center with complaints of severe chest pain and palpitation that had started immediately after he had eaten his dinner. He underwent urgent coronary angiography with possible diagnosis of acute coronary syndrome. His coronary arteries were found to be normal. Since his symptoms were partially relieved, he was discharged. He was referred to our hospital 3 days later, with aggravation of the same complaints. On admission, his abdominal physical examination was normal except for mild epigastric tenderness. His respiratory sounds were diminished in his left lower hemithorax. His pulse rate was 112/minute and blood pressure was 90/50mmHg. His body temperature was 37.5°C. He had sinus rhythm in electrocardiography (ECG). A hemogram and biochemical test results were as follows: white blood cell count 17,280/mm^3^ (4.8 to 10.8mm^3^), urea 65mg/dL (5 to 23mg/dL), creatinine 4.2mg/dL (0.6 to 1.3mg/dL), total bilirubin 3.5mg/dL (0.2 to 1.2mg/dL), gamma-glutamyl transpeptidase 78U/L (12 to 64U/L), aspartate transaminase 105U/L (5 to 34U/L), alanine transaminase 118U/L (0 to 55U/L), and C-reactive protein 314mg/dL (0.0 to 5.0 mg/dL). A CT examination was performed to exclude aortic aneurysm or dissection. There was pleural fluid in both sides; an air bubble containing pericardial fluid reaching to 2cm thickness was noted. There was minimal free fluid in perihepatic, perisplenic and retrovesical spaces. With these findings and clinical situation, severe mediastinitis was considered, and his detailed clinical history was taken into consideration once again. The patient later told that he had eaten chopsteak (meat on the bone) in his dinner just before the onset of his symptoms. Then the CT images were checked again, and a dense 2cm-long linear foreign body was found in the posterior aspect of his left atrium (Figure 1).

**Figure 2:**
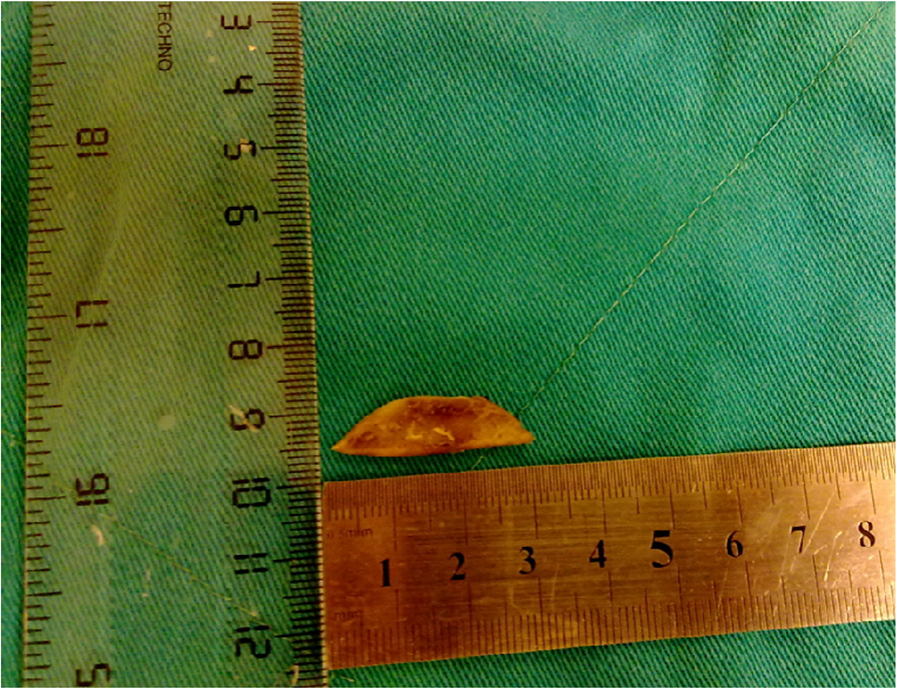
**Surgically removed bone fragment.**

These findings revealed a perforation of esophagus by a foreign body, and the patient underwent urgent left thoracotomy. At surgery, a sharp contoured piece of bone was found, perforating the 1/3 distal aspect of the posterior wall of his esophagus. It reached to the pericardium, penetrating it and eroding his left atrium. The adventitia of the aorta was affected on the left. There was pus in his pericardium. The foreign body was removed (Figure 2). After abundant serum irrigation, his esophagus was repaired by primary sutures, and pleura was attached on the defect side and supported by tissue adhesives. Finally, a percutaneous jejunostomy was performed for feeding by laparotomy. Parenteral broad spectrum antibiotic therapy and feeding from the jejunostomy was started. No postoperative complication was observed, and the patient was discharged from our hospital on the 15th day. After 3 days, he was readmitted and hospitalized again with the complaints of fever and weakness. Contrast extravasation from the primary suture zone was found in a CT examination, then he underwent endoscopic stent placement. His clinical condition deteriorated rapidly due to mediastinitis and he died because of sepsis and multiorgan failure.

## Discussion

Esophagus perforations due to foreign bodies have high mortality and morbidity rates. Diagnosis can be made by detailed clinical history, physical examination and CT. Immediate diagnosis and early treatment are important in preventing high mortality and morbidity in a case of esophagus perforation. The treatment depends on preventing and controlling sepsis and infection, and maintaining the continuity of digestive tract and nutrition. While early intervention performed within the first 24 hours of injury offers favorable outcomes, delayed surgery has increased mortality and morbidity rates [4]. In our case, his first complaints were confused with acute coronary syndrome in the previous hospital; therefore, the patient underwent coronary angiography. He was discharged because of normal findings in coronary angiography and partial relief of chest pain. The aggravation of his chest pain and his admission to our hospital took 3 days. The main reason for his mortality may be due to this delayed diagnosis and treatment. It is crucial that a clinician inform the radiologist about a patient’s clinical history and situation to establish a rapid and true radiological diagnosis. In our patient, although mediastinitis was diagnosed, the radiologist did not indicate the etiology in the lack of clinical history. After sufficient information, he revisited the CT and was able to demonstrate the bone piece and set the diagnosis of esophagus perforation. In our case, the most prominent cardiac symptoms including chest pain and palpitation were due to the perforation of esophagus and pericardium, and close relation of the bone piece to the left atrium and aorta. There was no sign of arrhythmia in the preoperative ECG. In cases of esophagus perforations or foreign bodies, a right or left thoracotomy decision is made according to the level of esophageal disease. While right intercostal thoracotomy is more convenient for 2/3 proximal esophagus lesions, left lower thoracotomy should be performed for 1/3 distal lesions. There is a publication that suggests the benefits of strengthening the defect or wound by pleural, pericardial or intercostal muscle patch grafts in addition to primary suturing [5]. Mediastinal pleural space must be debrided and irrigated and drainage is also important [5]. In our case, we preferred primary repair which was supported by pleural autograft. Despite developments in diagnosis and treatment of esophagus perforations, mortality rates still remain above 20% [6]. If there is no delay in diagnosis, the patient might survive.

## Conclusions

Sometimes, a piece of bone can cause esophagus perforation and injure the pericardium and myocardium. Lack of knowledge of detailed clinical history may cause confusion because of the similarity between the symptoms of acute coronary syndrome and those of esophagus perforation. Because of this, the patient’s detailed clinical history should always be checked and an appropriate medical imaging technique (that is, CT) chosen. Early proper diagnosis and treatment may reduce the mortality rate.

## Consent

Written informed consent was obtained from the patient’s next of kin for publication of this case report and any accompanying images. A copy of the written consent is available for review by the Editor-in-Chief of this journal.

## Abbreviations

CT: Computed tomography
ECG: Electrocardiography

## References

[1] Basagiannis C, Spartalis E, Karagkiouzis G, Panagoulias G, Tomos P. Successful surgical management of severe mediastinitis caused by a perforating esophageal foreign body. Hippokratia. 2012;16:93. PMC3738406 23930070

[2] Sharland MG, McCaughan BC. Perforation of the esophagus by a fish bone leading to cardiac tamponade. Ann Thorac Surg. 1993;56:969–71. 10.1016/0003-4975(93)90368-r 8215678

[3] Caplin JL, Franks R, Poole-Wilson PA. An unusual traumatic cause of cardiac tamponade and mitral regurgitation. Clin Cardiol. 1984;7:109–11. 10.1002/clc.4960070207 6705293

[4] Shaker H, Elsayed H, Whittle I, Hussein S, Shackcloth M. The influence of the ‘golden 24-h rule’ on the prognosis of esophageal perforation in the modern era. Eur J Cardiothorac Surg. 2010;38:216–22. 10.1016/j.ejcts.2010.01.030 20304664

[5] Byaruhanga R, Kakande E, Mwambu T. A rare case of a patient with a foreign body in the esophagus for two years which perforated into the mediastinum. Afr Health Sci. 2012;12:569–71. 10.4314/ahs.v12i4.28 PMC3598303 23513078

[6] Chirica M, Champault A, Dray X, Sulpice L, Munoz-Bongrand N, Sarfati E, et al. Esophageal perforations. J Visc Surg. 2010;147:117–28. 10.1016/j.jviscsurg.2010.08.003 20833121

