# Correction to: Esophagus perforation and myocardial penetration caused by swallowing of a foreign body leading to a misdiagnosis of acute coronary syndrome: a case report

**DOI:** 10.1186/s13256-018-1654-7

**Published:** 2018-03-31

**Authors:** Uysal Erdal, Dokur Mehmet, Kirdak Turkay, Ikidag A. Mehmet, Nacak Ibrahim, Bakir Hasan

**Affiliations:** 1 Department of General Surgery and Transplantation Center, Sanko University Hospital, Gaziantep, Turkey; 2 Department of Emergency, Sanko University Hospital, Gaziantep, Turkey; 3 Department of General Surgery, Uludag University School of Medicine, Bursa, Turkey; 4 Department of Radiology, Sanko University Hospital Gaziantep, Gaziantep, Turkey; 5 Department of Thoracic Surgery, Sanko University Hospital, Gaziantep, Turkey; 6 Department of General Surgery, Sanko University Hospital, Gaziantep, Turkey

## Correction

In the publication of this article [1], the Acknowledgements section was missing.

Acknowledgements

We would like to thank Dr. Ahmet Orhan Gurer for his help during the notification process.

This has now been included in this correction.

## References

[1] Erdal U, Mehmet D, Turkay K, et al. Esophagus perforation and myocardial penetration caused by swallowing of a foreign body leading to a misdiagnosis of acute coronary syndrome: a case report. J Med Case Rep. 2015;9:57. 10.1186/s13256-015-0532-9 PMC4363457 25889407

